# Motor recovery by the aberrant pyramidal pathway in a patient with cerebral infarct

**DOI:** 10.1097/MD.0000000000020282

**Published:** 2020-05-29

**Authors:** Sung Ho Jang, Jun Lee, You Sung Seo

**Affiliations:** a Professor, Department of Physical Medicine and Rehabilitation, College of Medicine, Yeungnam University, Department of Physical Medicine and Rehabilitation, College of Medicine, Yeungnam University 317-1, Daemyungdong, Namku, Taegu, Republic of Korea; b Professor, Department of Neurology, College of Medicine, Yeungnam University; c Department of Physical Medicine and Rehabilitation, College of Medicine, Yeungnam University, Department of Physical Medicine and Rehabilitation, College of Medicine, Yeungnam University 317-1, Daemyungdong, Namku, Taegu, Republic of Korea.

**Keywords:** aberrant pyramidal tract, cerebral infarct, diffusion tensor tractography, motor recovery, transcranial magnetic stimulation

## Abstract

**Introduction:**

The aberrant pyramidal tract (APT) refers to the collateral pathway of the pyramidal tract (PT) descending through the medial lemniscus in the midbrain and pons. We report on a patient who showed changes of the APT from the early stage to the chronic stage concurrent with motor recovery following an infarct in the cerebral peduncle.

**Patient concerns:**

An 84-year-old female patient presented with moderate motor weakness of her upper and lower extremities (2^+^/2^+^) due to cerebral infarct on the right cerebral peduncle of midbrain. One week after her stroke, her motor weakness was similar to that at the onset (2^+^/2^+^). During 5 weeks’ rehabilitation, her motor weakness recovered to the point that she was able to move upper and lower extremities against gravity with some resistance (4^-^/4^-^).

**Diagnosis:**

Cerebral infarct on the right cerebral peduncle of midbrain

**Interventions:**

She participated in a comprehensive rehabilitative management program, including movement therapy, neurotrophic drugs, and neuromuscular electrical stimulation therapy of the left finger extensor and ankle dorsiflexor muscles.

**Outcomes:**

On 1-week and 6-week diffusion tensor tractographys (DTTs), the right PT was not reconstructed, but the right APT, which descended through the medial lemniscus pathway at the midbrain and pons and the pyramid at the medulla, was observed. The right APT became thicker on six-week DTT compared with 1-week DTT. On 1-week transcranial magnetic stimulation study, an motor evoked potential with delayed latency and decreased amplitude was evoked from the affected (right) hemisphere (latency: 24.4 msec and amplitude: 0.2*u*V). In contrast, its latency was decreased and amplitude was increased on six-week transcranial magnetic stimulation study (latency: 21.8 msec, amplitude: 0.8 *u*V)

**Conclusions:**

We demonstrated changes in the APT from the early stage to the chronic stage concurrent with motor recovery in a patient with an infarct in the cerebral peduncle. Our findings have important implications that a spared APT could contribute to the motor recovery in patients with cerebral infarct when the PTis completely injured at the onset of cerebral infarct,.

## 1 Introduction

The pyramidal tract (PT) is the major neuronal tract for mediation of voluntary movements in the human brain.^[1,2]^ The PT has various collateral pathways in the human brain, and 1 such collateral pathway, the aberrant PT (APT), descends through the medial lemniscus at the midbrain and pons after separating from the original PT at the midbrain.^[3–6]^ Diffusion tensor tractography (DTT), which is derived from diffusion tensor imaging (DTI), allows visualization and estimation of the PT and APT in the live human brain.^[4,7–12]^ DTT exams have demonstrated that the APT can act as a motor recovery mechanism in patients with brain injury.^[7,8,10–13]^ However, its role in the motor recovery mechanism is not clearly elucidated.

In this study, we report on a patient whose APT changed from the early stage to the chronic stage concurrent with motor recovery following an infarct in the cerebral peduncle.

## 2 Case report

**Figure 1:**
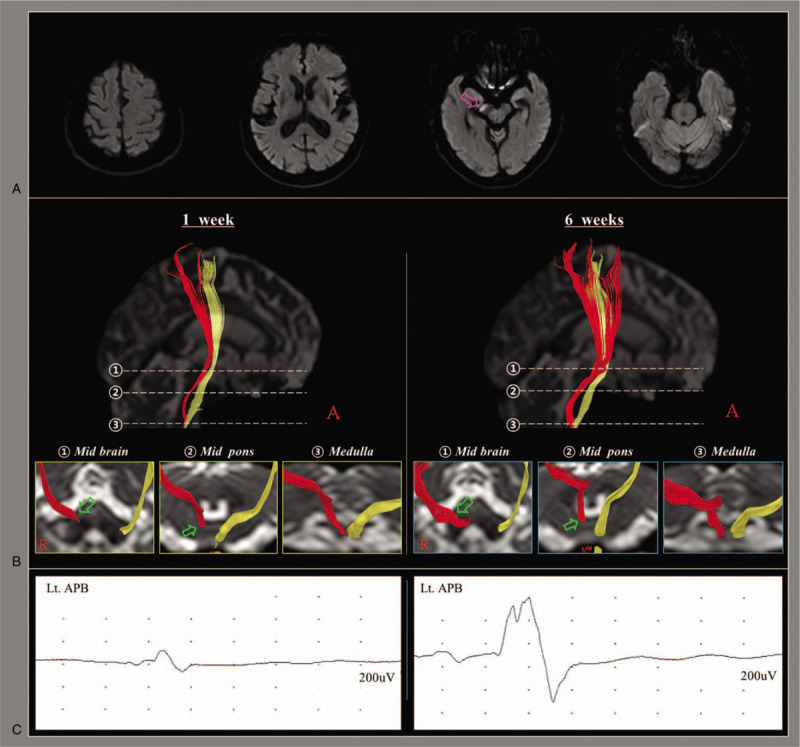
(A) Diffusion-weighted images at the onset show an infarct in the right cerebral peduncle of mid brain (pink arrow). (B) Results of diffusion tensor tractography (DTT). The right aberrant pyramidal tract (APT) descends through the medial lemniscus pathway (green arrows) at the midbrain (1) and pons (2), and the pyramid (3) at the medulla. The right APT became thicker on 6-wk DTT comparing with 1-wk DTT. (C) Results of transcranial magnetic stimulation (TMS). On 1-wk TMS study, motor evoked potential with delayed latency and decreased amplitude is evoked from the right hemisphere (latency: 24.4 ms and amplitude: 0.2*u*V). The latency decreased and amplitude increased on 6-wk TMS study (latency: 21.9 ms, amplitude: 0.8 *u*V). APT = aberrant pyramidal tract, DTT = diffusion tensor tractography, TMS = transcranial magnetic stimulation.

An 84-year-old right-handed female patient presented with moderate motor weakness of her upper and lower extremities (2^+^/2^+^) due to a cerebral infarct on the right cerebral peduncle of midbrain (Fig. 1-A). After conservative management for the cerebral infarct at the department of neurology of a university hospital, she was transferred to the rehabilitation department at the same university hospital 1 week after onset. Her motor weakness persisted at a level similar to the onset (2+/2+). She participated in a comprehensive rehabilitative management program, including movement therapy, neurotrophic drugs (amantadine: 300 mg, ropinirole: 1.5 mg, and levodopa: 750 mg), and neuromuscular electrical stimulation therapy of the left finger extensor and ankle dorsiflexor muscles. Over 5 weeks’ rehabilitation, her left motor weakness recovered to the point that she was able to move upper and lower extremities against gravity with some resistance (4-/4-). At 6 weeks after onset, she was able to grasp and release an object using her left hand and walk independently on an even floor. Patient has provided informed consent for publication of the case and the study protocol was approved by the institutional review board of the university hospital.

## 3 Diffusion tensor tractography

DTI data were acquired twice (1 week and 6 weeks after onset) using a 1.5T (Philips Medical Systems, Best, The Netherlands) with single-shot echo-planar imaging. Imaging parameters were as follows: acquisition matrix=96 × 96; reconstructed to matrix=192 × 192; field of view=240 × 240 mm^2^; TR= 10,398ms; TE= 72ms; parallel imaging reduction factor=2; EPI facto = 59; b = 1000 s/mm^2^; and a slice thickness of 2.5 mm. Each DTI replication was intra-registered to the baseline “b0” images to correct for residual eddy-current image distortions and head motion effect, using a diffusion registration package (Philips Medical Systems). Fiber tracking used the fiber assignment continuous tracking algorithm implemented within the DTI task card software (Philips Extended MR Work Space 2.6.3). Three regions of interest (ROIs) were placed for the PT on the color map (blue: superior-inferior orientation, red: medio-lateral orientation, green: antero-posterior orientation). The first ROI was placed on the posterior limb of internal capsule and the second ROI was placed on the PT area of the anterior medulla (portion of anterior blue color) and additional ROI was placed on the isolated APT area (behind the posterior transpontine fiber (red) at the pontine level).^[4]^ Fiber tracking was initiated at the center of any voxel with a fractional anisotrophy (FA) of <0.2 and a tract turning angle of <60 degrees. Values of FA and tract volume (TV) for the APT were measured.

On 1-week and six-week DTTs, the right PT was not reconstructed, but the right APT, which descends through the medial lemniscus pathway at the midbrain and pons and the pyramid at the medulla, was observed. The right APT became thicker on six-week DTT compared with 1-week DTT (Fig. 1-B). In addition, DTT parameters of the right APT changed as follows: 1 week: FA: 0.47 and TV: 996, and six weeks: FA: 0.44 and TV: 1291.

## 4 Transcranial magnetic stimulation (TMS)

TMS was performed using a Magstim Novametrix 200 magnetic stimulator with a 9 cm mean diameter circular coil (Novametrix Medical Systems Inc, Wallingford, CT, USACortical stimulation was performed with the coil held tangentially over the vertex. The circular coil was positioned flat on the scalp with its center over Cz (international 10/20 system). A counterclockwise current provided stimulation to the left hemisphere, and the right hemisphere was stimulated by a clockwise current. Motor evoked potentials (MEPs) were obtained from both abductor pollicis brevis muscles in a relaxed state. Magnetic stimulation was applied at 100% of maximal output. Each site was stimulated 3 times with an interval of at least 10 seconds between stimulation. The shortest latencies and average peak-to-peak amplitudes were calculated from the data obtained.

On 1-week TMS study, an MEP with delayed latency and decreased amplitude was evoked from the affected (right) hemisphere (latency: 24.4 msec and amplitude: 0.2*u*V). In contrast, its latency was decreased and amplitude was increased on six-week TMS study (latency: 21.8 msec, amplitude: 0.8 *u*V)(Fig. 1-C).

## 5 Discussion

In this study, we observed DTT and TMS in a patient with cerebral infarct who recovered motor abilities and whose APT changed during five weeks’ rehabilitation. We believe that the motor weakness in this patient recovered by changes of the spared APT that existed at the onset for the following reasons. First, we could observe the APT on one-week DTT. We assume that the APT might exist from the onset because the patient showed similar (moderate) weakness at 1 week after onset compared with the onset. A previous study reported that the APT was observed in about 17.9% of the hemispheres in normal subjects on DTT.^[9]^ Second, the DTT parameters changed: increment of TV (996→1291) without significant change of FA value (0.47→0.44). FA value indicates the degree of directionality of water diffusion and the integrity of white matter microstructures such as axon, myelin, and microtubule.^[14–16]^ TV is determined by the number of voxels included in a neural tract, thereby suggesting the total number of fibers of a neural tract.^[16]^ Therefore, the increment of TV in the APT on 6-week DTT indicates an increment in fiber number of the APT compared with 1-week DTT. Third, MEP parameters changed: the increment of amplitude (0.2uV→0.8uV) with decrement of the latency (24.4 msec → 21.8 msec). The latency of a MEP indicates the fastest velocity of a neural tract and amplitude suggests the total amount of neural fibers of the PT.^[17]^ Therefore, the results of the later TMS suggest facilitation of the APT during 5 weeks’ rehabilitation. The mildly delayed latency (24.4 ms) of 1-week MEP appeared to coincide with those of APTs in previous studies (25.6 ms, 24.0 ms).^[11,12]^

Since the introduction of DTI, several studies have reported that the APT was attributed to motor recovery in patients with brain injury.^[7,8,10–13]^ Since 2009, several case studies have reported on the APT in patients who recovered from severe weakness following an infarct in the pontine basis or corona radiata, traumatic or spontaneous intracerebral hemorrhage in the corona radiata using DTT, and functional MRI or TMS.^[7,8,11,12]^ Lindenberg et al [2010] demonstrated in 35 chronic stroke patients using DTT that the patients who had alternative motor fibers that might be APT showed better motor function than the patients lacking those alternative motor fibers.^[10]^ Compared with studies that demonstrated APT change at a chronic stage following brain injury, this study demonstrated APT changes from an early stage to chronic stage after the onset of a cerebral infarct. However, limitations of DTT should be considered. First, DTT can produce false negative results throughout the white matter of the brain because of crossing fiber or partial volume effect.^[18]^ Second, this study is limited because it is a case report.

In conclusion, we demonstrated APT changes from the early stage to the chronic stage using DTT and TMS concurrent with motor recovery in a patient with an infarct in the cerebral peduncle. Our findings have important implications, that even when the PT is completely injured at the onset of cerebral infarct, a spared APT could contribute to the motor recovery in patients with cerebral infarct. Further complementary DTT studies involving larger case numbers are required.

## Author contributions

Sung Ho Jang: Study concept and design, Manuscript development and writing, Jun Lee: Study concept and design, Manuscript editing, You Sung Seo: Study concept and design, Acquisition and analysis of data, Manuscript authorization

**Conceptualization:** Sung Ho Jang, You Sung Seo.

**Data curation:** You Sung Seo.

**Methodology:** Lee Jun.

**Writing – original draft:** Sung Ho Jang, You Sung Seo.

**Writing – review & editing:** Sung Ho Jang, You Sung Seo.
